# Prevalence, clinical characteristics, and biological markers of postmenopausal osteoporosis and knee osteoarthritis in Beijing: study protocol for a cross-sectional and prospective study

**DOI:** 10.3389/fmed.2025.1582533

**Published:** 2025-06-04

**Authors:** Zhenkai Lu, Wenye Feng, Yaxing Wang, Xuming Zhang, Xu Wei, Ming Chen, Shangquan Wang, Ting Cheng, Xin Cui, Yanming Xie

**Affiliations:** ^1^Institute of Basic Research in Clinical Medicine, China Academy of Chinese Medical Sciences, Beijing, China; ^2^Wangjing Hospital, China Academy of Chinese Medical Sciences, Beijing, China

**Keywords:** postmenopausal osteoporosis, knee osteoarthritis, cross-sectional study, prospective, follow-up, risk factors

## Abstract

**Background:**

Postmenopausal osteoporosis (PMOP) and knee osteoarthritis (KOA) are common musculoskeletal disorders that share risk factors such as aging, hormonal changes, and inflammation. PMOP often remains asymptomatic until fractures occur, while KOA leads to pain and disability. Their comorbidity remains underexplored, particularly in community settings. This study aims to investigate the prevalence, risk factors, and clinical features of PMOP-KOA comorbidity among Beijing residents aged 45–80 years, providing evidence-based recommendations for early identification, prevention, and management.

**Methods:**

Over 2 years, this study will conduct a PMOP-KOA screening and prospective follow-up in the Beijing community to investigate the incidence, risk factors, and clinical characteristics of PMOP-KOA. This study will undertake bone mineral density detection, collect biological samples, and record information via questionnaires.

**Discussion:**

The study aims to investigate the incidence, risk factors, and clinical characteristics of PMOP-KOA comorbidity and explore related traditional Chinese medicine syndromes based on large community-based samples in Beijing. Data on PMOP-KOA occurrence and associated risk factors over the 2 years follow-up will be available on the Chinese Clinical Trial Registry (ChiCTR2300073575).

## 1 Introduction

Postmenopausal osteoporosis (PMOP) and knee osteoarthritis (KOA) are common age-related degenerative musculoskeletal disorders. As society has aged, they have become significant health concerns, particularly for postmenopausal women, affecting both physical and mental wellbeing (1). PMOP and KOA share similar pathological mechanisms, including abnormal bone metabolism in subchondral bone and age-related changes (2, 3). The comorbidity of KOA and PMOP may have interconnected effects on bone mineral density (BMD) and bone metabolism, with KOA patients typically exhibiting increased systemic BMD, while PMOP patients experience decreased BMD (4). This opposing trend in BMD changes may be attributed to KOA’s influence on bone formation and PMOP’s promotion of bone resorption, highlighting the metabolic heterogeneity in PMOP-KOA patients. The complexity of bone metabolism changes in these two conditions, along with the lack of clear early diagnostic biomarkers, remains a major challenge, making the clinical management of comorbidity more difficult.

In recent years, an increasing number of countries have started to be more concerned about community-based public health programs (5–7). Typically, there is a large population with KOA or PMOP in the community, which are undiagnosed before fractures occur, meaning that community-based health programs for PMOP and KOA screening could be of great significance to residents. Studies have shown that most KOA patients also suffer from osteoporosis, with approximately one in five osteoporosis patients concurrently diagnosed with osteoarthritis (8, 9). However, epidemiological findings on PMOP, KOA, and other potential influencing factors vary across different regions (10). Additionally, a lack of follow-up is common in the majority of community-based studies.

This prospective study will investigate conventional risk factors and relevant symptoms as reported in traditional Chinese medicine in populations with a high risk of PMOP (11, 12). Based on the findings of this preliminary research, we will then conduct a further cross-sectional survey and a prospective follow-up study in the Beijing community. This prospective study aims to investigate the prevalence of PMOP and KOA, explore associated risk factors, and provide evidence-based recommendations for their prevention and management. Additionally, the study will conduct transcriptomic analyses on samples from different subtypes of PMOP-KOA patients to identify differentially expressed genes (DEGs) with high specificity and sensitivity. These DEGs will serve as potential biomarkers for PMOP-KOA subtyping, offering valuable molecular insights into disease classification and paving the way for personalized treatment strategies. This research on “Beijing Community Postmenopausal Osteoporosis and Knee Osteoarthritis Screening: a cross-sectional and prospective Study (BEYOND)” will provide reliable data for the prevention of PMOP-KOA among community-dwelling residents in Beijing area.

## 2 Materials and methods

**FIGURE 1:**
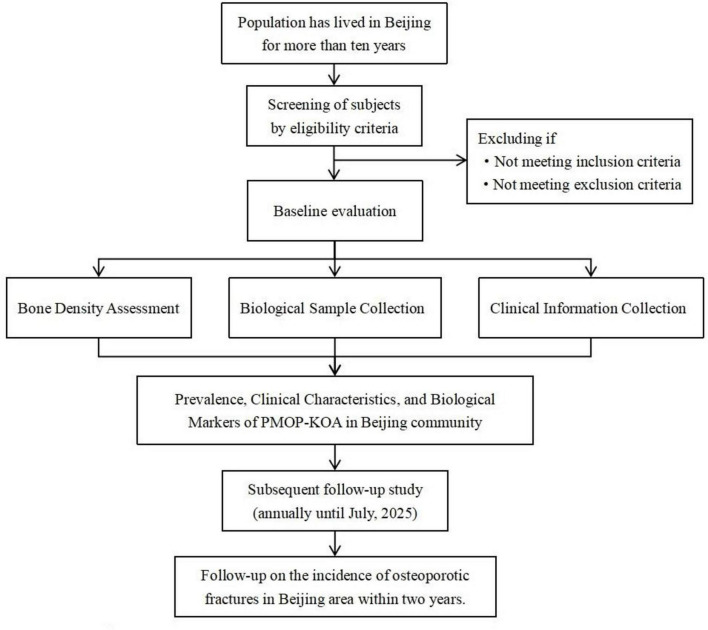
Study flow.

This is a community-based cross-sectional and prospective follow-up study. Through a preliminary literature review of both Chinese and English databases, we found that research on PMOP-KOA remains unstandardized, lacking support from multicenter, large-sample epidemiological studies. There is still a gap in understanding the clinical characteristics of patients with both conditions, as well as the genetic differences among PMOP-KOA patients with distinct traditional Chinese medicine (TCM) syndrome classifications. To address these gaps, we will recruit residents from 12 communities, applying predefined inclusion and exclusion criteria to screen participants for an epidemiological investigation. Data collection will include bone mineral density measurements, biological samples, and questionnaire-based information. Each participant will be followed for at least 2 years to track disease progression in PMOP-KOA patients. A flowchart of the research process is shown in Figure 1.

### 2.1 Participant recruitment

Due to the significant geographic and socioeconomic variations across different communities in Beijing, and considering the potential impact of economic status on bone mineral density, this study will recruit participants from Chaoyang District and Fengtai District. This approach aims to minimize potential biases related to educational background, work environment, and economic conditions.

Within these two administrative districts, 3–5 communities will be randomly selected. Local committees representing community residents or healthcare services will then be contacted. We will collaborate with community staff to post recruitment advertisements in public spaces, outlining the study’s objectives, content, significance, and potential benefits for participants.

### 2.2 Ethics and registration

This study has received approval from the Medical Ethics Committee of Wangjing Hospital, China Academy of Chinese Medical Sciences (Approval No.: WJEC-KT-2023-010-P002) and has been registered with the Chinese Clinical Trial Registry (Registration No.: ChiCTR2300073575). Prior to participation, all individuals or their legal representatives will be fully informed about the study and required to sign an informed consent form, adhering to the principles of the Helsinki Declaration.

### 2.3 Diagnostic criteria

According to the World Health Organization (WHO) and the Chinese Guidelines for the Diagnosis and Treatment of Primary Osteoporosis (13), the diagnosis of OP is primarily based on BMD measurements using dual-energy X-ray absorptiometry (DXA). The diagnostic criterion for OP, based on DXA measurements of axial bones (lumbar spine L1-L4, femoral neck, or total hip) or the distal one-third of the radius, is a T-score ≤ −2.5. According to the Chinese Guidelines for the Diagnosis and Treatment of KOA (14), the diagnostic and KL grading criteria for KOA are detailed in Table 1.

**TABLE 1:** Diagnostic criteria.

**OP**	
T-score ≥ −1.0	Normal
−2.5 < T-score < −1.0	Low bone mass
T-score ≤ −2.5	Osteoporosis
T-score ≤ −2.5 + Fragility fracture	Severe osteoporosis
**KOA**	
① Recurrent knee joint pain within the past month	KOA diagnosis: satisfies criterion 1+ any 2 of criteria 2, 3, 4, or 5.
② X-ray (standing position or weight-bearing position) shows joint space narrowing, subchondral bone sclerosis, and/or cystic changes, with osteophyte formation at joint margins	
③ Age ≥ 50 years	
④ Morning stiffness lasting ≤ 30 min	
⑤ Presence of crepitus during movement	
**Kellgren & Lawrence (KL) grading**	
No changes (normal)	0
Joint space appears normal (doubtful osteoarthritis)	I
Possible joint space narrowing (mild osteoarthritis)	II
Definite joint space narrowing with sclerosis (moderate osteoarthritis)	III
Severe joint space narrowing with significant sclerosis and bone deformities (severe osteoarthritis)	IV

Traditional Chinese medicine syndromes represent specific stages in KOA or PMOP progression, influenced by both internal and external factors. TCM-based treatment considers an individual’s constitution, environmental conditions, and seasonal changes, following the principle of “treatment based on syndrome differentiation.” This approach is fundamental in TCM, involving the identification of syndromes through symptom analysis and physical examination methods such as inspection, auscultation, olfaction, and palpation. By classifying patients based on syndrome differentiation, TCM provides a framework for stratifying individuals and selecting appropriate treatments tailored to distinct clinical presentations.

According to the Guidelines for the Diagnosis and Treatment of Osteoarthritis (2018 edition) and the Guidelines for the Diagnosis and Treatment of Primary Osteoporosis (2022 edition) issued by the China Association of Chinese Medicine, the primary TCM syndromes commonly observed in patients with KOA and PMOP include yang deficiency, yin deficiency, and combined yin-yang deficiency. In this study, syndrome classification will be conducted by two independent TCM practitioners with senior titles based on a standardized TCM four-diagnostic information collection form (inspection, listening/smelling, inquiry, palpation). In cases of disagreement, a third senior practitioner will be consulted for arbitration. Inter-rater reliability will be assessed using Cohen’s kappa coefficient to ensure diagnostic consistency. Participants will then be classified accordingly, and peripheral blood samples will be collected for high-throughput sequencing analysis.

### 2.4 Inclusion and exclusion criteria

The inclusion criteria for this study are as follows: (1) Participants must have resided locally for more than 10 years; (2) Participants must meet the diagnostic criteria for KOA, with a KL grade of II or higher (unilateral or bilateral), and simultaneously fulfill the diagnostic criteria outlined in the Guidelines for the Diagnosis and Treatment of Primary Osteoporosis for comorbid osteoporosis; (3) Participants must be postmenopausal women aged 50–65 years (50 ≤ age < 65); (4) Participants must not have taken estrogen or medications affecting bone metabolism (e.g., glucocorticoids, antiepileptic drugs, aluminum-containing antacids, proton pump inhibitors, chemotherapy drugs, anticoagulants, or thiazolidinediones) within the past month; (5) Participants must voluntarily enroll in the study, sign an informed consent form, and ensure that the consent process fully adheres to ethical principles.

The exclusion criteria are as follows: (1) Participants with physical disabilities that severely limit mobility or the ability to comply with study procedures, as assessed by clinical investigators; (2) Participants with other autoimmune diseases; (3) Participants with serious heart, liver, or kidney comorbidities; (4) Participants who are pregnant or lactating; (5) Participants who have received immunosuppressive therapy within the last 3 months; (6) Participants who are unwilling or unable to complete follow-up.

To minimize recall bias in fracture reporting, all self-reported fracture events must be confirmed through radiographic evidence (X-ray or CT report); cases without objective imaging confirmation will be excluded from fracture-related analysis.

### 2.5 Sample size

Through multiple clinical pilot investigations and expert consultations, a standardized participant information collection form has been developed and refined. Additionally, based on a comprehensive review of prior literature and expert discussions, this study has preliminarily identified the characteristic clinical symptoms and physical signs of PMOP-KOA. To estimate the required sample size, the Kendall method was initially applied, multiplying the number of variables by five, yielding a minimum sample size of 150 participants (15). To further enhance methodological rigor, a supplementary power analysis was conducted. Assuming a medium effect size (Cohen’s d = 0.5), a significance level (α) of 0.05, and a power (1–β) of 0.80, the required sample size for detecting group differences was calculated to be approximately 128 participants using standard formulas. Allowing for a 15% attrition rate due to potential loss to follow-up or incomplete data, the final target sample size was set at 150 participants. This dual approach ensures both practical feasibility and statistical robustness.

### 2.6 Bone density assessment

A HOLOGIC Wi dual-energy X-ray absorptiometry (DEXA) scanner (United States) will be utilized to measure bone density at the lumbar spine and bilateral hips, recording the T-score and Z-score at each site. The instrument’s precision will be maintained at 1%, ensuring that the error in repeated measurements remains below 1%. Daily calibration and maintenance will be conducted by trained professionals to ensure measurement accuracy and optimal device performance.

### 2.7 Biological sample collection

Blood samples will be collected from participants categorized by the three TCM syndrome classifications using PAXgene™ Blood RNA Tubes (16), with 2.5 mL of venous blood obtained per individual. Immediately after collection, samples will be stored at 4°C and subsequently transferred to a −80°C freezer on the same day following a stepwise freezing protocol. To minimize potential batch effects, all collected samples will be randomly assigned to RNA extraction and sequencing batches. Furthermore, RNA quality will be assessed using the RNA Integrity Number (RIN), and only samples with RIN ≥ 7 will be included in downstream analyses. All samples will be transported to the BGI Biological Sample Testing Center via dry ice cold chain logistics for subsequent RNA-seq high-throughput sequencing (17).

The same batch of blood samples used for RNA-seq will also be used for RT-qPCR validation. RNA (1 μg) will be used as a template for reverse transcription to synthesize complementary DNA (cDNA) (RK20403; ABclonal, Woburn, MA, United States). Target gene sequences will be obtained from the GenBank database, and primers will be designed using PrimerExpress Software v2.0. GAPDH will be selected as the internal reference gene, and its stability across different experimental conditions will be verified using geNorm and NormFinder algorithms. Experimental results will be analyzed using the relative quantification method, with 2^–ΔΔCt^ as the indicator to represent the relative gene expression levels.

### 2.8 Clinical information collection

All participants will complete a face-to-face paper questionnaire and undergo a comprehensive physical examination. The questionnaire and examination will cover general characteristics, menstrual history, menopausal age, pregnancy and childbirth history, education and income levels, smoking and drinking habits, medical and medication history, fall frequency, dietary habits, and TCM syndrome classification. Detailed information on fractures, including frequency, causes, locations, and treatments, will also be collected.

Based on relevant standards and guidelines, a preliminary TCM information collection form for PMOP-KOA participants was developed. Through multiple clinical pilot investigations and expert consultations, the questionnaire was continuously revised and refined, leading to the final information collection form and participant screening form. Additionally, the following scales will be used to assess physical activity levels and quality of life: International Physical Activity Questionnaire (IPAQ) (18), International Osteoporosis Foundation (IOF) 1-Minute Osteoporosis Risk Assessment Test (19), EuroQol 5-Dimension (EQ-5D) Instrument (20). Both the IPAQ and EQ-5D have undergone cultural adaptation and validation in Chinese populations in previous studies. Reliability analysis in similar cohorts reported Cronbach’s α values greater than 0.80, indicating good internal consistency. For this study, only validated Chinese versions of the IPAQ and EQ-5D will be used to ensure measurement accuracy and cultural relevance.

### 2.9 Follow-up study

A subsequent follow-up study will be conducted annually until July 2025. Participants who miss two or more consecutive scheduled assessments will be considered lost to follow-up. To minimize dropout, the study team will implement strategies such as regular reminder calls or messages, flexible scheduling of follow-up visits, and providing modest incentives. For handling missing data due to loss to follow-up, multiple imputation techniques will be used under the assumption that data are missing at random. Sensitivity analyses will be conducted to evaluate the robustness of results to different missing data mechanisms.

### 2.10 Primary outcomes

This study will be conducted in two phases. In the cross-sectional phase, the primary outcomes will include BMD and participants’ clinical information, which will be used for TCM classification and subsequent differential gene expression analysis. During the follow-up period, the incidence of osteoporotic fractures will be the most critical outcome. While self-reported data and interviews will provide valuable information on newly occurring fractures, all reported fracture cases will be immediately reassessed using DEXA to verify bone loss and confirm fracture events. Participants who experience fractures will be asked to provide detailed information, including the time and cause of the fracture, the affected site, and post-fracture treatment received. In cases of uncertainty, medical records and imaging reports will be reviewed to ensure accuracy.

### 2.11 Data management and statistical analysis

Data entry and logical verification were performed by two independent personnel using an electronic management system (National Copyright Registration Number: 2023SR0693141) to handle missing and abnormal values, ensuring data accuracy. Quality control measures were established in accordance with the requirements of clinical epidemiological cross-sectional studies and the specific characteristics of TCM research (21, 22). For standardization, all knee X-ray imaging and BMD assessments for participants from all community sub-centers were conducted at Beijing Electric Power Hospital. All recorded data must be preserved as original records and must not be altered arbitrarily.

Statistical analysis was performed using R 4.3.1. Continuous variables are presented as mean ± standard deviation ($\bar{\text{x}}$ ± s), while categorical variables are reported as percentages. Fisher’s exact test or Chi-square test was used for between-group comparisons. Variance estimation was performed using Taylor series linearization and replication methods. A two-sided *P*-value < 0.05 was considered statistically significant.

To control for potential confounding variables—such as body mass index (BMI), physical activity, dietary habits, and history of estrogen treatment multivariable regression models (e.g., logistic regression or Cox proportional hazards models for time-to-event data) will be employed. In addition, propensity score matching (PSM) or inverse probability of treatment weighting may be used where appropriate to balance covariates among comparison groups and enhance causal inference validity.

## 3 Discussion

Postmenopausal osteoporosis-KOA presents a complex clinical challenge, due to the overlapping pathophysiological mechanisms of both conditions (23, 24). OP is characterized by low bone mass and microstructural deterioration, leading to increased fracture risk (25, 26); KOA, in contrast, contributes to joint degeneration and functional impairment (27–29). The coexistence of these two conditions may compound effects on bone metabolism, thereby influencing disease progression and clinical outcomes. Despite growing interest in community-based musculoskeletal health research, epidemiological data on PMOP-KOA comorbidity remain limited, particularly in Beijing. This study aims to address these gaps by systematically investigating the prevalence, clinical characteristics, and risk factors of PMOP-KOA.

Through a cross-sectional design with a large sample size, this research explores TCM syndrome differentiation, functional activity, and metabolic indicators in PMOP-KOA patients. The follow-up phase will track osteoporotic fracture incidence and assess the long-term impact of risk factors via dynamic monitoring. The integration of high-throughput sequencing provides additional insights into potential molecular biomarkers, offering a novel perspective on disease classification and personalized management.

However, this study has several limitations. First, recruitment limited to specific Beijing communities may reduce the generalizability of findings to broader populations. Second, real-world variability in age distribution and sex ratios may introduce bias, potentially affecting clinical applicability. Third, reliance on self-reported medical history and medication use introduces recall bias, which may compromise data accuracy. To address these issues, future research will consider expanding participant recruitment to multiple centers across different geographic regions to improve population representativeness. Additionally, incorporating electronic medical records and objective clinical data will help mitigate recall bias. A longitudinal study design with repeated measures and the integration of biological markers is also planned to enhance causal inference and the robustness of findings. Despite these limitations, the current study provides a foundational dataset for improving early identification and management strategies for PMOP-KOA patients, thereby contributing to better healthcare planning and targeted interventions in community settings.
